# Naivety dies with the calf: calf loss to human hunters imposes behavioral change in a long-lived but heavily harvested ungulate

**DOI:** 10.1186/s40462-024-00506-5

**Published:** 2024-09-23

**Authors:** Lukas Graf, Henrik Thurfjell, Göran Ericsson, Wiebke Neumann

**Affiliations:** 1 Department of Wildlife, Fish, and Environmental Studies, Swedish University of Agricultural Sciences, Skogsmarksgränd, 901 83 Umeå, Sweden; 2 Southern Swedish Forest Research Centre, Swedish University of Agricultural Sciences, Sundsvägen 3, 234 22 Lomma, Sweden; 3 Swedish Species Information Centre, Swedish University of Agricultural Sciences, Alma Allé 8E, 756 51 Uppsala, Sweden

**Keywords:** *Alces alces*, Anti-predator behavior, Hidden Markov Model, Integrated Step Selection Function, Sweden

## Abstract

**Background:**

In prey, patterns of individual habitat selection and movement can be a consequence of an individuals’ anti-predator behavior. Adjustments of anti-predator behavior are important for prey to increase their survival. Hunters may alter the anti-predator behavior of prey. In long-lived animals, experience may cause behavioral changes during individuals’ lifetime, which may result in altered habitat selection and movement. Our knowledge of which specific events related to hunting activity induce behavioral changes in solitary living species is still limited.

**Methods:**

We used offspring loss in a solitary and long-lived ungulate species, moose (*Alces alces*), as our model system. We investigated whether offspring loss to hunters induces behavioral changes in a species subjected to heavy human harvest but free from natural predation. To test for behavioral change in relation to two proxies for experience (calf fate and age), we combined movement data from 51 adult female moose with data on their offspring survival and female age. We tested for adjustments in females’ habitat selection and movement following calf harvest using Hidden Markov Models and integrated Step Selection Analysis to obtain behavioral state specific habitat selection coefficients.

**Results:**

We found that females with a harvested calf modified habitat selection and movement during the following hunting season. Female moose selected for shorter distance to roads during the night, selected for shorter distance to forests and greater distance to human settlements following calf harvest than females who had not lost a calf. The survival of twins in a given hunting season was related to female age. Older females we more likely to have twins survive the hunting season.

**Conclusions:**

Our findings suggest that losing offspring to human harvest imposes behavioral changes in a long-lived ungulate species, leading to adjustments in females' habitat selection and movement behavior, which may lower the risk of encountering hunters. In our study, female moose that experienced calf loss selected for lower distance to forest and selected for greater distance to human settlements during periods of high hunting pressure compared to females without the experience of calf loss during the previous hunting season. We interpret this as potential learning effects.

**Supplementary Information:**

The online version contains supplementary material available at 10.1186/s40462-024-00506-5.

## Introduction

The behavioral traits of animals are innate or learned through experience [1, 2]. In prey species, anti-predator behavior is vital for increasing both individual and offspring survival, thereby contributing to individuals’ fitness [3]. Efficient anti-predator behavior demands flexibility and development that allows prey to adjust to their major source of mortality. This suggests that an individual’s experiences throughout its lifetime shape anti-predator behavior [4–6]. The expression of anti-predator behaviors can differ between individuals across species’ geographical ranges according to predator occurrence, thereby affecting individuals’ likelihood of survival under the risk of predation [7], as well as the species’ evolutionary success [3, 8].

Generally, anti-predator behavior addresses the predation risk of the most abundant predator or the predator causing the most fatalities at a given time and place, including human hunters [4, 9]. Animals across different taxa have been found to adjust their behavior in accordance to varying degrees of predation risk, which has been interpreted as potentially learning from predation attempts [10, 11]. In systems with intensive human harvest, and where humans act as the most dominant predator, the impact of hunters can overshadow the effect of natural predators on prey response [4, 12, 13].

In general, different species have shown indications of learning processes or memory formation (e.g., roe deer (*Capreolus capreolus*), [14, 15]), as well as in terms of adjusted anti-predator behavior (e.g., in bettongs (*Bettongia lesueur*) [16] or great tits (*Parus major*) [17]). Specifically, some behavioral changes suggest learning effects may be triggered by experiencing mortal events of a conspecific, group member, or offspring (e.g., in damselfish (*Acanthochromis polyacanthus*) [18], crows (*Corvus brachyrhynchos*) [19] or common goldeneyes (*Bucephala clangula*) [20]). In species subjected to human harvest or predation, individuals may adjust their behavior in relation to hunting activity [6, 21], as well as towards carnivore species (e.g., Brown bears (*Ursus arctos*) [22] or wolves (*Canis lupus*) [23]). Despite the known capability of individuals to adjust their behavior towards the mortality risk during the hunting season [6, 24, 25], our knowledge of the mechanisms underlying this change in anti-predator behavior is still limited, particularly for solitary-living species. Similarly, we are limited in measuring the behavioral responses involved.

Ungulates are among the most harvested species groups worldwide [26, 27]. The risk of human harvest invokes the need for prey to modify their behavioral responses towards human disturbances, especially during the hunting season [9, 28, 29]. For example, hunting of ungulates is usually only allowed during daytime and human hunters often utilize areas near roads [24], making their spatiotemporal distribution predictable. For prey species that are subject to human harvest, behavioral changes have to match the spatiotemporal risk for harvest (i.e. minimizing the risk of encountering hunters by hiding or moving less [12]). For example, human-habituated and gregarious reindeer (*Rangifer tarandus*) developed new behaviors once they started to be culled [30], and solitary-living roe deer (*C. capreolus*) modified their behavior in relation to hunting regimes [31, 32]. Furthermore, experiencing mortality events of conspecifics may cause individuals to alter their behavior, which in return may increase their own survival changes [6, 31, 33, 34]. In short, we still lack a comprehensive understanding of which events related to nonlethal experiences that may lead to behavioral adjustments in long-lived solitary species and so far mostly proxies for experience, such as age, have been used [6].

In this study, we investigated whether offspring loss to human harvest results in adjusted anti-predator behavior in terms of habitat selection and movement. As our model system, we used adult Swedish female moose in two populations, which are heavily harvested. In our study areas, hunting accounts for most of the mortality, as large carnivores are functionally extinct [35, 36]. Importantly, female moose live mostly solitary and within the Swedish hunting policy, calves must be harvested before their mothers, potentially generating several opportunities throughout the females’ approximate 20-year lifetime to test whether females adjust their behavior after experiencing calf harvest [37]. Furthermore, this policy framework generates a “life insurance” for females accompanied by calves, and potential double learning events for females with twins.

In ungulates, movement and habitat selection are major factors that can define an individuals’ fate and are generally closely linked to the surrounding environment [38, 39]. Further, the spatial behavior and movement of an individual may depend on the presence of offspring [40–42]. Recent research has highlighted that considering behavior-dependent habitat selection (e.g., is the animal foraging or explorative) improves the estimation and prediction of animal habitat preferences and advances our understanding of ecology [43, 44]. To test for individual adjustments in anti-predator behavior, we analyzed movement- and behavior-specific habitat selection in adult female moose following the harvest of their calves. Specifically, we investigated differences in females’ behavioral-state-specific habitat selection and movement in relation to two proxies for experience, as we expected change of anti-predator behavior in response to harvest risk and female experience to be dependent on the behavioral state an animal is in [6, 25].

We tested our hypothesis that *female moose that lose a calf to human harvest will adjust their state-specific habitat selection and movement in the following hunting season (in an effort to evade human hunters).* Hence, our null hypothesis is that *no behavioral adjustment occurs following calf loss to human harvest*.

First, we predict (a) that female moose exhibit non-naive anti-predator behavior (i.e., anti-predator behavior is adjusted to evade human hunters) after losing a calf (a specific experience) during the previous hunting season. We expect female to select habitats farther away from human infrastructure (i.e. roads), stay more sheltered and reduce movement following calf loss. Second, we predict (b) that changes in state-specific behavior in relation to age (as a proxy for and measure of experience) will show similar trends as they do for calf fate. Finally, we predict (c) that older females have a greater chance of having their calves survive the hunting season as they are more experienced and have adapted non-naive anti-predator behaviors.

## Materials and methods

### Study area

**Fig. 1:**
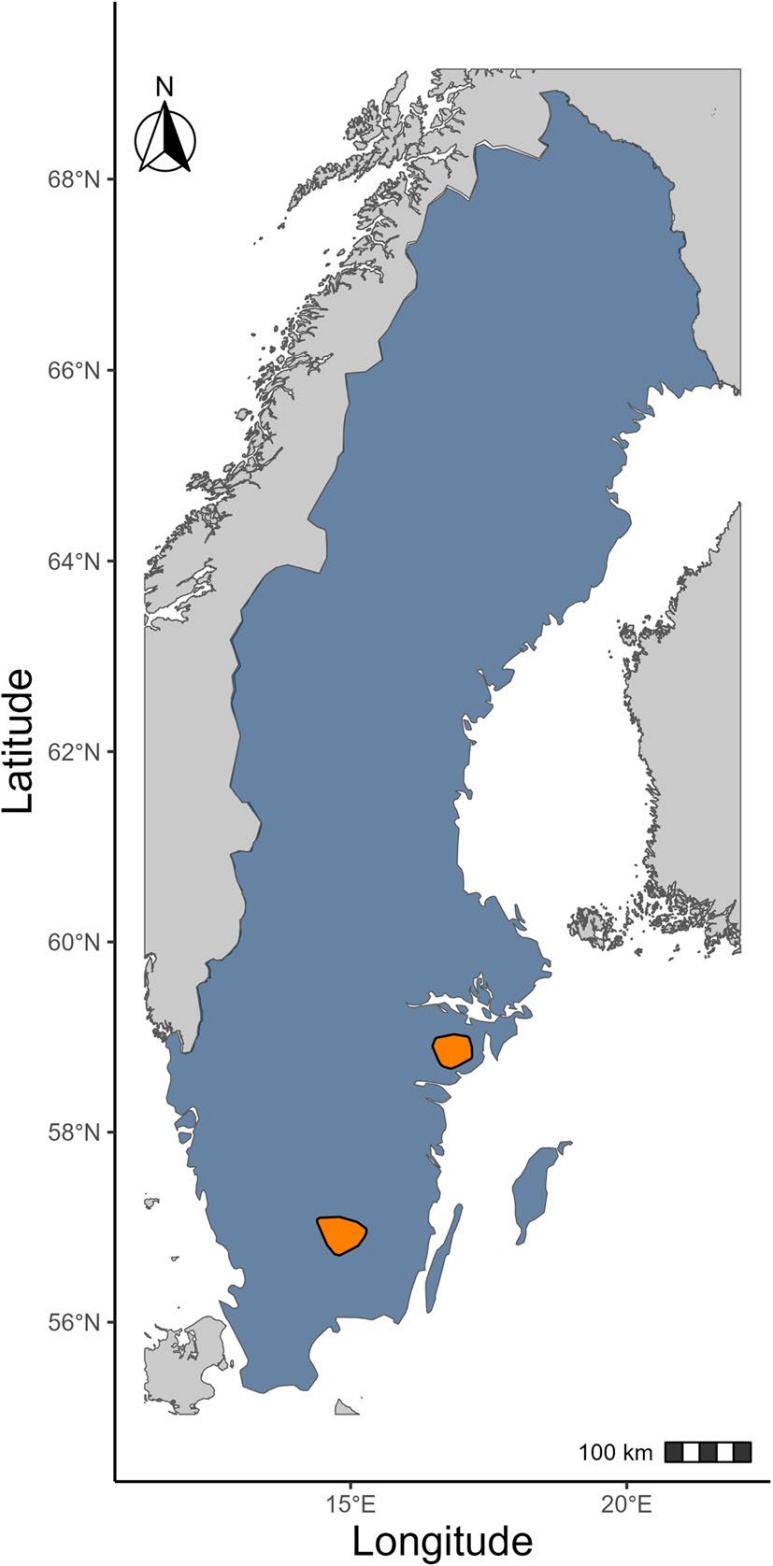
The two study areas (orange) as the minimum convex polygon around all GPS positions in southern Sweden. Fennoscandia is marked in gray, and Sweden is marked in blue

Our study combined data from two sites in the southern boreal region (56° N, 14° E and 58° N, 17° E) (Fig. 1). At both sites, mixed forests as patches of deciduous forests (e.g., birch (*Betula sp.*), aspen (*Populous sp.*), elm (*Ulmus glabra*), oak (*Quercus robur*), maple (*Acer platanoides*)) and coniferous forest such as Scots pine (*Pinus sylvestris*) and Norway spruce (*Picea abies*) intermix with agricultural fields in a flat to gently rolling terrain. In our study areas, the annual moose hunting season starts after the moose rut on the second Monday in October and ends in February [45, 46]. Moose hunting is usually carried out in a hunting team and with the use of baying dogs [45, 47]. Hunting pressure is generally highest during the first 3 weeks of the hunting season and decreases thereafter. The majority of the moose harvest occurs from the second Monday of October until the end of December [48].

### GPS data

We analyzed 10 years of Global Positioning System (GPS) positions of 51 female moose individuals from 2008 to 2018. Moose were tranquilized from a helicopter with a dart-gun that injected a mixture of etorphine-acepromazine and xylazine in winter (January/February) [49]. Moose were equipped with GPS Plus Collars following our standard protocol [50, 51]. The GPS-collars were programmed to a 3-h sampling rate. We estimated the individual age from tooth wear [37, 50]. To cover major parts of the hunting season and the period before the annual moose hunts start, we analyzed moose GPS data from the 1st of August to the end of December (when hunting pressure decreases significantly), leaving in total 5 months of GPS data to analyze in each year.

### Calf survival and reproduction data

We assessed the number of calves born and their summer survival by field observations during two periods, shortly after birth and before the hunting season, following our standard protocol [52]. A calf was defined as lost if the calf was absent from the mother on two consecutive observations before and after the hunting season in a given reproductive year. We verified calf survival during the hunting season with field observations at the end of the hunting season and complemented them by hunters reporting if they shot either the calf of the collared female. Therefore, we considered any calf loss during the hunting season as a calf loss to hunters. To analyze the potential behavioral differences in female moose during the hunting season after calf loss, we matched the GPS data in a given year with information on calf loss in the previous hunting season. We omitted GPS data of moose individuals from the analysis in years when we could not verify during any visual calf check whether she had a calf at heel. We omitted this data to avoid inducing further noise in the analysis, as behavioral patterns of female moose with and without offspring can differ [40–42]. We did not consider the amount of calves an individual had lost over its collaring period, as the age at capture strongly varied between individuals and older individuals could have lost many calves before being fitted with a GPS collar, which would introduce bias and uncertainty. Given the lack of information on lost calves a given female may have experienced during her lifetime, we decided to use a more conservative approach. To analyze behavioral changes in relation to experience (calf fate in the previous hunting season), we chose to compare behavior between calf fate groups instead of using the accumulated number of calves lost. This approach enabled us to account for differences in recent experience (as measured by calf fate) in the previous hunting season and compare the difference in behavior among females. In total, we analyzed GPS data from 128 individual moose years of 51 female moose individuals; the average tracking time was 2.51 ± 1.57 years.

### Statistical analysis

#### Segmentation of movement behavior

Habitat selection of ungulates can be behavior-specific (i.e., animals select different habitats based on their behavioral state [6, 43, 53]), making the incorporation of behavioral states into our analysis a vital departure point for understanding animal responses to the risk of predation [43]. To obtain movement-specific habitat selection coefficients and test for individual-based behavioral change following calf harvest, we first fitted a two-state Hidden Markov Model (*HMM*). We performed a segmentation of our movement data in order to avoid three-way and four-way interactions between movement parameters, habitat, hunting pressure and calf fate or age in later sections of the analysis.

**Fig. 2:**
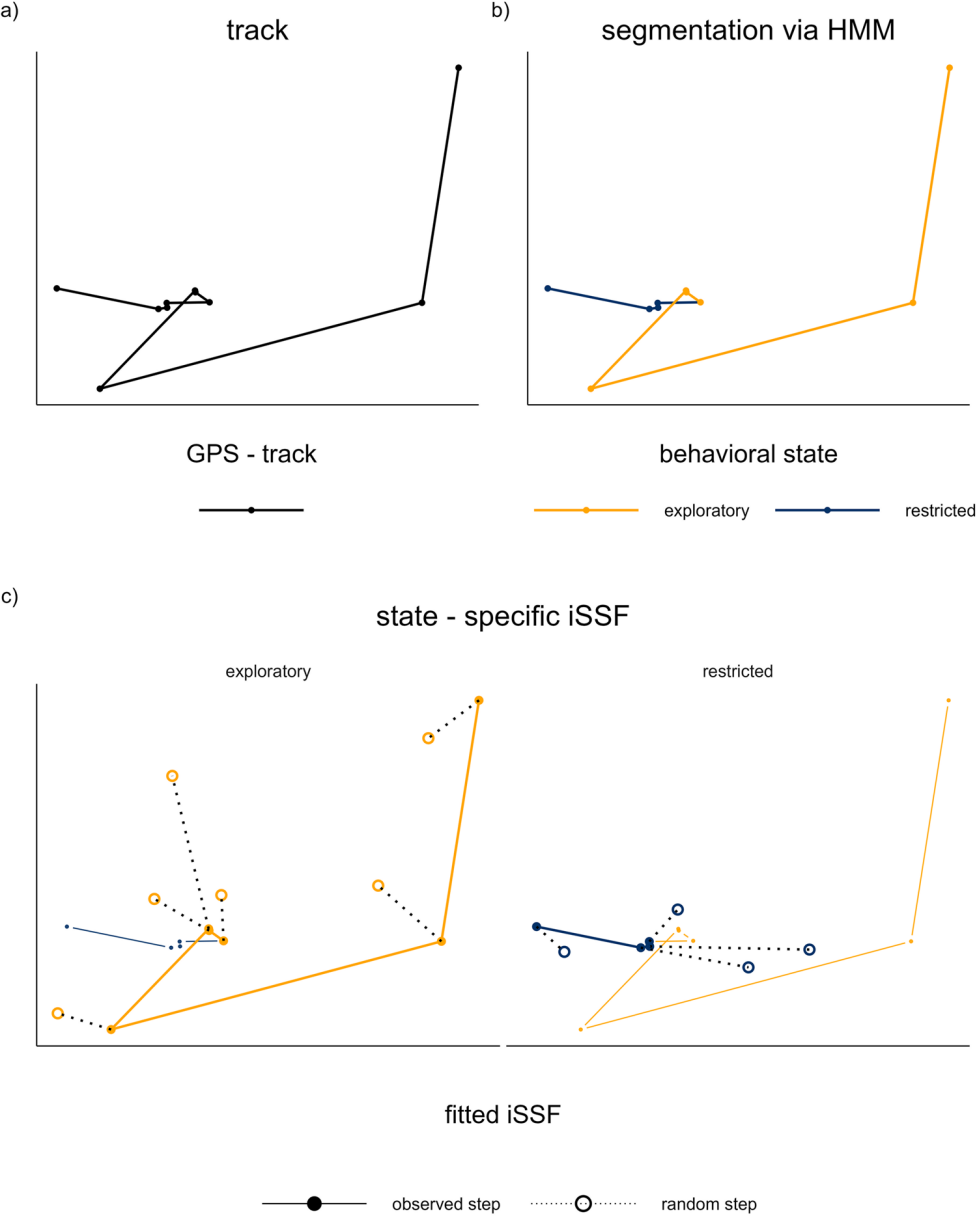
**a**–**c** Our methodological approach to analyzing the state-specific habitat selection of female moose. **a** The GPS track of an animal, **b** the segmentation into behavioral states according to the Hidden Markov Model (HMM) (see “Segmentation of movement behavior” section), where yellow points and lines indicate an exploratory behavioral state and blue points and lines indicate a restricted behavioral state. **c** The fitted integrated Step Selection Function (iSSF) (see “Analysis of habitat selection” section), where dashed lines and hollow points indicate the alternative steps available to the animal at a given observed step. For visualization purposes, we only show one of 25 available random steps in (**c**)

We fitted the two-state *HMM* with two data streams using the *momentuHMM*—package [54]. We segmented the movement trajectories of each female moose into two unobserved behavioral states (*restricted* and *exploratory)*, which we expected to differ in the degree an individual exposes itself and thus may affect its encounter risk with hunters [25, 55, 56] (see Fig. 2a, b). The unobserved states were estimated from step length and turning angles. We considered the restricted behavioral state reflect lower movement rates and thus generally more cautious behavior in terms of habitat selection. Further, we assumed the restricted state to reflect foraging behavior and a low chance of encountering human hunters. In contrast, we expected the exploratory behavioral state to reflect higher movement rates with high directional persistence and transitioning behavior, potentially with higher risk behavior due to higher probability of encountering human hunters [6, 33].

Initial step length parameters were chosen according to Neumann et al. [57] to account for bimodal movement patterns of moose over a 3-h time interval. The restricted state was defined with an initial mean step length of 100 m (± 100 m SD and 0.001 zero mass parameter (to account for a low probability of no movement)), a mean turning angle of π, and a turning angle concentration of 0.001. Next, we defined the exploratory behavioral state with longer step lengths and high directional persistence. The exploratory state had an initial mean step length of 450 m (± 450 m SD, 0.001 zero mass parameter), a mean turning angle of 0.1, and a turning angle concentration of 0.99. We modeled step lengths using a gamma distribution and turning angles using a Von Mises distribution. We had to fit a zero mass parameter, as we had few steps in our dataset where animals did not move between GPS—locations [54]. We only defined two states and did not test for evidence of further states (e.g., encamped behavioral state) since we considered the spatial resolution (25 × 25 m) of our environmental covariates too coarse (see “Analysis of habitat selection” section) to model a behavioral state with extremely low movement rates. We closely followed procedures of previous applications of *HMMs* within ecological research [25] to fit the two-state *HMM* in our analyses*.* We then applied the Viterbi algorithm to our dataset to assign each step the most likely behavioral state based on the *HMM* [54, 56].

#### Analysis of habitat selection

To analyze state-specific habitat selection and movement of female moose (see “Segmentation of movement behavior” section) we fitted integrated Step Selection Functions (*iSSF*) [25, 58]. We split the data based on year and behavioral state and fitted separate *iSSFs* to analyze state-specific habitat selection on an annual basis. For each observed step, we generated 25 alternative steps [58–60]. We fitted *iSSF*s using the *amt*—package [61], using a gamma distribution to fit step lengths and a Von Mises distribution to fit turning angles to generate alternative steps [58] (Fig. 2c). We added one meter to all step lengths to account for all steps with no movement, as this would have led to errors when fitting the gamma distribution.

The behavior of big game (i.e., ungulates) hunters makes the spatiotemporal harvest risk for prey predictable in relation to infrastructure (e.g., hunting close to roads [24, 62, 63]) or cover. Based on our experience of spatial behavior of our study species, knowledge of behavior of Swedish hunters and previous literature on anti-predator behavior towards human hunters, we matched the used and available steps of the *iSSF* with three environmental covariates. We chose distance to roads, human settlements and forests as they play a crucial role in the spatial interactions of human hunters and moose [6, 24, 33, 45]. We calculated distance to forest using the Swedish Land Survey map from a binary raster with a 25 × 25 m spatial resolution [64], updated with data on clear cuts from the Swedish Forest Agency [65]. Distance to human settlements was calculated from the Swedish Land Survey map from a binary raster [64]. Distance to roads was calculated from a binary raster from the Swedish Transport Agency [66]. We expected selection for greater distance to forest to represent riskier behavior. For distance to human settlements and distance to roads it was the opposite, here we considered selecting for shorter distance to roads or human settlements to reflect riskier behavior. Covariates were extracted at the end of each step. Next, we added information on harvest risk as indexed by different hunting pressures (*categorical*, three factors: no hunting pressure (1st of Aug to the first Sunday of October, the day before the hunting season starts); high hunting pressure (2nd Monday of October (first day of the hunting season) and the following 3 weeks when hunting pressure is highest [48]) and low hunting pressure (after high pressure ends until the end of December)). Further, we added time of day (*categorical*, two factors: day and night) to account for diurnal patterns in habitat selection and no hunting during the night (Table 1). Time of day was calculated using the *time_of_day()*—function in the *amt*—package [61]. All environmental covariates were z-score transformed at the individual level [67].

**Table 1:** Overview of the covariates used in this study

Covariate	Type	Transformed	Impact	Prediction	References
Distance to forest	Continuous	Yes	Selecting for habitats outside of forests increases visibility to human hunters	(a), (b)	[6]
Distance to roads		Yes	Selecting for habitats close to roads is a risk	(a), (b)	[6, 24]
Distance to human settlements		Yes	Selecting for habitats close to human settlements is a risk	(a), (b)	[63]
Step length	Continuous	No*	Higher movement increases visibility and chance to encounter hunters	(a), (b)	[6, 33, 58]
Turning angles		Yes	–		[58]
Time of day	Factor with two levels (day, night)	No	Human hunters are limited by light to hunt, selection for riskier areas will take place during the night	(a), (b)	[6, 33, 63]
Hunting pressure	Factor with three levels (none, high hunting pressure, low hunting pressure)	No	Different hunting pressure will cause prey to adjust their behavior to counterbalance human hunters	(a), (b)	[6, 30, 33]
Calf fate	Binary	No	Calf loss is expected to cause behavioral change	(a), (c)	[6, 11, 34, 52]
Age	Continuous	No	Older females are more experienced and adjust movement and habitat selection	(b), (c)	[6, 52]

*We fitted the natural logarithm of step length to all step selection models

We analyzed the behavioral state-specific *iSSFs* by fitting individual conditional logistic regressions for each animal in each year, using the matched sets of used and available steps as the response variable [58, 68]. Conditional logistic regressions were fit using the *survival*—package [69]*.* We included one-way interactions between both factors (time of day, hunting pressure) and environmental covariates in all models. We further fitted step length, the natural logarithm of step length and the cosine of the turning angles as covariates to account for the underlying movement process in the *iSSF*. As we fitted a model for every individual in each year and behavioral state, we obtained 256 models. The *β* coefficients estimated in models for the restricted behavioral state would therefore reflect habitat selection and movement with a lower probability of human hunter encounters but also more cautious behavior, while coefficients of the exploratory behavioral state would reflect a higher probability of hunter encounters and generally less cautious behavior.

#### Testing for change in habitat selection and movement

We applied a two-step approach to summarize coefficients of the individual models and draw inference at the population level. The two-step approach allowed us to analyze differences in *β* coefficients of individual models in relation to age and calf fate in a second modeling step to account for among- and within-individual variation [60, 70]. We chose to account for individual variation using the two-step approach instead of a mixed model approach, as the structure of the mixed model would have been very complex and we were interested in testing for differences in the individuals’ behavior [71]. Further, the two-step approach enabled us to avoid complex models with three-way to four-way interactions between variables that are difficult to interpret (e.g., *distance to forest* × *hunting pressure* × *calf fate* × *age*). The mixed effect model also likely would have led to convergence issues and would have complicated drawing inference about the same parameter sets and interactions for both behavioral states and both calf fate groups.

To ensure that we could analyze calf fate and female age within the same model and we could draw inference from both variables, we tested whether calf fate was not correlated to moose age using the Mann‒Whitney U test. We summarized *β* coefficients and tested for effects of calf fate (*calf loss* vs *no calf loss* in the previous hunting season) and age on habitat selection at the population level by using inverse-variance weighted (*IVW*) regression [32, 72]. The *IVW* regression gives less weight to parameters with higher variance, reducing uncertainty when drawing population level inference*.* We used the *β* coefficient sets (*β*_distance to forest_, *β*_distance to forest × night_, etc.) obtained from the conditional logistic regressions in each behavioral state as the response variables and fitted and ranked several competing sets of *IVW* models using Akaike Information Criterion corrected for small sample sizes (AICc). We averaged models with a Δ—AICc < 2 [73]. We included calf fate and age, as well as a two-way interaction between age and calf fate, as predictors in the *IVW* regressions to test for behavioral changes (see Table 2 for the ecological interpretations between variables and interactions). This resulted in 24 different sets of competing candidate models to analyze behavioral differences, one for each *β* coefficient set in each behavioral state.

**Table 2:** Overview of the fitted models used to test for differences in β coefficients and estimated-observed mean movement from the iSSF

Model	Formulation	Effect
Null	y ~ 1	Habitat selection and movement remain unchanged throughout lifetime
Age	y ~ age	Habitat selection and movement change in relation to age as a proxy for experience, but change is unrelated to calf fate
Calf fate	y ~ calf fate	Calf loss induces behavioral change in habitat selection and movement, regardless of age
Full	y ~ calf fate × age	Both age and calf fate influence habitat selection and movement

All models with habitat selection coefficients as the response were fitted with the inverse variance (1/SE^2^) as a weight, models for movement (Δ—estimated mean step length—observed mean step length) were fitted as regular linear model

Similarly, we tested for effects of calf fate and age on movement by calculating the estimated step length [74] using the formula $l_{mean} = \frac{k + \beta \ln (l)}{{\theta^{ - 1} - \beta_{l} }}$, where *k* and *θ* determine the scale and shape of the observed gamma distribution, respectively; *β* and *β*_*ln(l)*_ are the estimated coefficients for the observed step length and natural logarithm of step length, respectively, of the conditional logistic regression [58]. We then subtracted the mean step length of each individual in each year and behavioral state. Like with habitat selection, we used AICc model selection to select the most parsimonious linear model describing the effects of age and calf loss on the Δ—estimated mean step length—observed mean step length in each behavioral state (Table 2, Appendix. Tables 7, 8).

To investigate whether experience of females increases calf survival in the *ongoing* hunting season (i.e. prediction (c)), we applied a generalized linear model with a binomial family using moose age and the number of calves before the hunting season as predictors and calf survival in the hunting season as the response (*binary*). Due to relatively small sample size for a given female (2.89 ± 1.97 SD years), we decided against a mixed modeling approach as we would not have a sufficient sample size for each individual, which would introduce bias [75, 76]. We included a two-way interaction between age and the number of calves. As we had no limitations regarding known calf loss status in the previous season and available data for the current season, the reproduction dataset was larger than the habitat selection dataset and included reproduction and survival data for 76 individual females in 218 moose years.

All the statistical analyses and data handling were conducted in R version 4.1.3 [77].

## Results

Out of our 51 females, 13 never experienced calf loss, whereas 14 experienced calf loss every year. The remaining 24 females had both experiences, i.e., years in which they lost a calf and years in which they did not lose a calf to human hunters. The Mann‒Whitney U test showed that calf loss to human hunters occurred at all ages (*W* = 7815.5, *p* = 0.833) (see Fig. 1 in Appendix).

### Model selection of calf fate and age

Including calf fate as a predictor of behavioral change in females’ habitat selection increased the model performance by 70.8% in all models. Calf fate had a greater impact on habitat selection in a restricted behavioral state than in an exploratory behavioral state (i.e., calf fate as a predictor of behavioral change improved 83.3% of the models for habitat selection in the restricted behavioral state, compared to 58.3% of the models for habitat selection in the exploratory behavioral state) (Table 3). In contrast, adding female age—as an additional proxy for experience—improved the overall model fit for 58.3% of the models. We found a greater impact of age on habitat selection in the exploratory behavioral state (50.0% of the models for habitat selection in restricted behavioral and 66.7% of the models for habitat selection in exploratory behavioral state; however, see Appendix Tables 1–6).

**Table 3:** Results of the IVW regression analysis of habitat selection of female moose (n = 51) from 2008 to 2018 in an exploratory and restricted behavioral state

β-habitat	Predictor for behavioral adjustment	HMM—state			
		Restricted		Exploratory	
		Estimate	SE	Estimate	SE
Distance to forest	Intercept	− 0.043	± 0.023	− 0.128	± 0.048
	Age	n.i.		− 0.006	± 0.006
	Calf fate (no loss)	− 0.048	± 0.033	n.i.	
	Calf fat (no loss) × age	n.i.		n.i.	
Distance to forest × high hunting pressure	Intercept	0.064	± 0.080	− 0.015	± 0.064
	Age	− 0.012	± 0.007	0.009	± 0.007
	Calf fate (no loss)	n.i.		− 0.063	± 0.046
	Calf fat (no loss) × age	n.i.		n.i.	
Distance to forest × low hunting pressure	Intercept	− **0.070**	** ± 0.030**	0.045	± 0.054
	Age	n.i.		− 0.008	± 0.007
	Calf fate (no loss)	**0.097**	** ± 0.042**	− 0.029	± 0.049
	Calf fat (no loss) × age	n.i.		n.i.	
Distance to forest × night	Intercept	0.047	± 0.027	0.056	± 0.086
	Age	n.i.		0.015	± 0.008
	Calf fate (no loss)	0.044	± 0.042	n.i.	
	Calf fat (no loss) × age	n.i.		n.i.	
Distance to human settlements	Intercept	− 0.049	± 0.138	− **0.075**	** ± 0.046**
	Age	0.014	± 0.021	n.i	
	Calf fate (no loss)	0.154	± 0.143	**0.165**	** ± 0.065**
	Calf fat (no loss) × age	n.i.		n.i.	
Distance to human settlements × high hunting pressure	Intercept	**0.287**	** ± 0.156**	− 0.015	± 0.083
	Age	n.i		− 0.006	± 0.015
	Calf fate (no loss)	− **0.461**	** ± 0.226**	− 0.053	± 0.094
	Calf fat (no loss) × age	n.i.		n.i.	n.i.
Distance to human settlements × low hunting pressure	Intercept	0.134	± 0.189	0.339	± 0.141
	Age	0.011	± 0.030	− 0.032	± 0.014
	Calf fate (no loss)	− 0.193	± 0.205	n.i.	
	Calf fat (no loss) × age	n.i.		n.i.	
Distance to human settlements × night	Intercept	− 0.169	± 0.128	0.005	± 0.050
	Age	0.008	± 0.021	n.i.	
	Calf fate (no loss)	0.050	± 0.141	− 0.031	± 0.088
	Calf fat (no loss) × age	n.i.		n.i.	
Distance to roads	Intercept	0.277	± 0.032	0.183	± 0.038
	Age	n.i.		− 0.004	± − 0.005
	Calf fate (no loss)	− 0.074	± 0.042	n.i.	
	Calf fat (no loss) × age	n.i.		n.i.	
Distance to roads × high hunting pressure	Intercept	0.058	± 0.103	0.006	± 0.026
	Age	− 0.001	± 0.016	n.i	
	Calf fate (no loss)	− 0.173	± 0.242	− 0.019	± 0.045
	Calf fat (no loss) × age	0.040	± 0.021	n.i	
Distance to roads × low hunting pressure	Intercept	0.029	± 0.060	− 0.044	± 0.061
	Age	− 0.004	± 0.010	0.008	± 0.008
	Calf fate (no loss)	n.i.		n.i.	
	Calf fat (no loss) × age	n.i.		n.i.	
Distance to roads × night	Intercept	− **0.343**	** ± 0.035**	− 0.312	± 0.029
	Age	n.i.		n.i.	
	Calf fate (no loss)	**0.104**	** ± 0.049**	0.033	± 0.048
	Calf fat (no loss) × age	n.i.		n.i.	

β-habitat denotes which habitat selection coefficients were tested for behavioral adjustments, while the predictor for behavioral adjustment shows the fitted covariates to the model. The estimate represents the estimated regression coefficient of the most parsimonious model for predicting the adjustment of habitat selection, and the SE represents the standard error. n.i. denotes “not included”, which indicates that a model containing this predictor had a Δ—AICc < 2 and was subsequently removed from the analysis. The effects of calf fate on behavior (SE not overlapping) are marked in bold

### Impact of calf fate on state-specific habitat selection

#### Habitat selection in the restricted behavioral state

Regardless of calf fate, females selected for shorter distance to forests when in a restricted behavioral state (Table 3). In addition, females that had lost a calf selected for shorter distance to forests compared to females that had not lost a calf during low hunting pressure (see also Fig. 2 in the Appendix for visual representation). During high hunting pressure, (the first 3 weeks of the moose hunting season), females that had lost a calf selected for greater distance to human settlements than females that had not lost a calf. Females in both calf fate groups selected for larger distance to roads before the hunting season, with females that had lost a calf selecting to be further away than females with surviving calves. Females who had experienced calf loss selected for shorter distance to roads during the night compared to those that had not lost a calf.

#### Habitat selection in the exploratory behavioral state

Before the hunting season, in the exploratory behavioral state, females selected for shorter distance to human settlements after a calf loss compared to females that had not lost a calf. This response changed with the onset of the hunting season. During low hunting pressure, females of both calf fate groups selected for greater distance to human settlements. When hunting pressure was low, we found no effect of calf loss on selection for distance to roads, regardless of behavioral state (*β* coefficient set *distance to roads* × *low pressure* in Table 3).

### Impact of age on state-specific habitat selection

#### Habitat selection in the restricted behavioral state

In a restricted behavioral state, we found that age had a general effect on habitat selection in female moose, regardless of the fate of their calf in the previous year. Older females selected for shorter distances to forests. Older females selected for greater distances to human settlements during both day and night. We found only weak adjustments of selection for distance to roads during both high and low hunting pressure.

#### Habitat selection in the exploratory behavioral state

In an exploratory behavioral state, before the hunting season and during low hunting pressure, females selected for shorter distance to forest as they became older. This pattern reversed under high hunting pressure but was modified by time of day. At night, we found an increase in selection of greater distances to forests for older females in an exploratory behavioral state, compared to daytime. Older females selected to be closer to human settlements when in an exploratory behavioral state and during times of high and low hunting pressure. Age affected the selection of habitats regarding their distance to roads, with older females selecting shorter distances to roads compared to younger females. During times of low hunting pressure, this pattern reversed and older females selected for greater distance to roads. Selection for distance to roads at night was not affected by age, only by calf fate, regardless of behavioral state.

### Impact of calf fate and age on state-specific movement

**Fig. 3:**
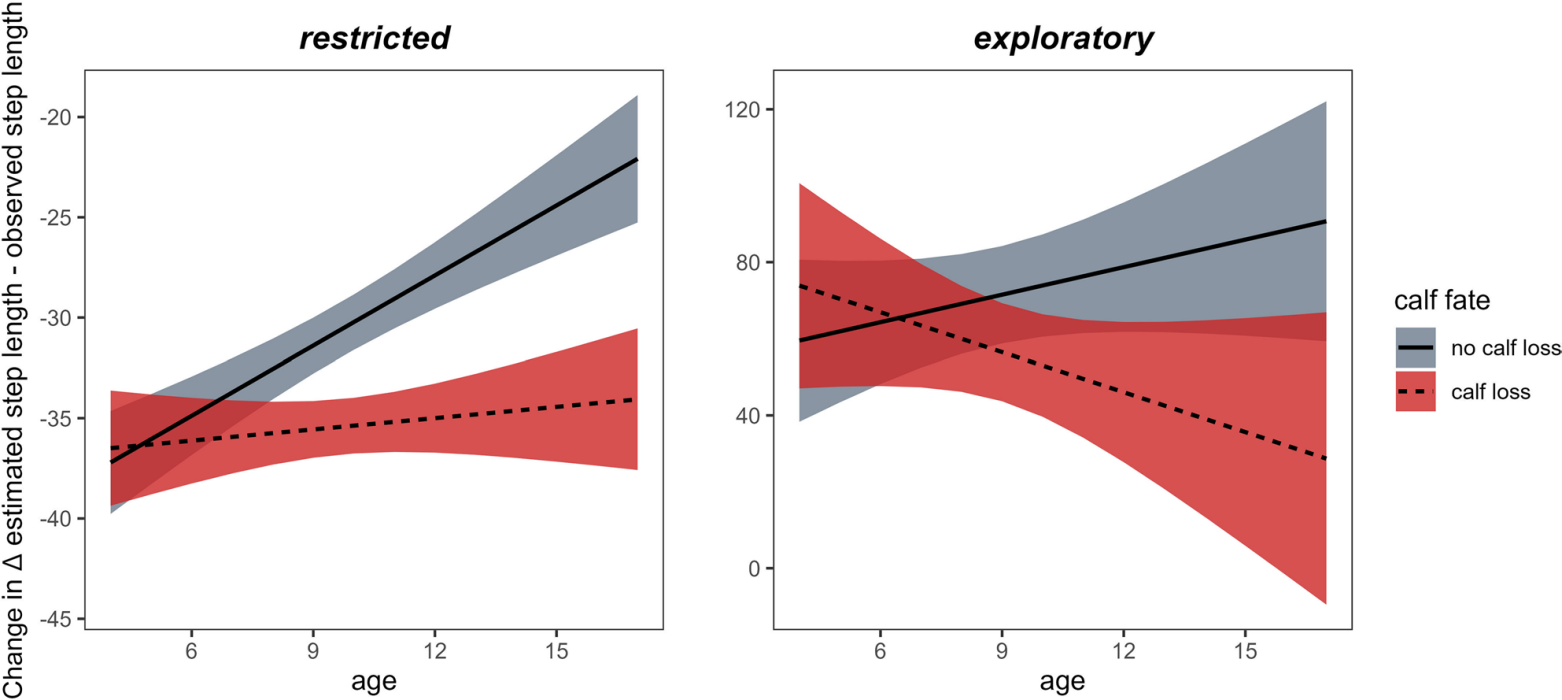
Effects of calf loss and aging on female (n = 51 females) movement on Δ estimated—observed step length estimated by the most parsimonious linear model for each behavioral state (restricted/exploratory). Red and gray shaded areas indicate estimated standard errors

According to the *HMM*, female moose showed a mean step length of 68.3 m (± 59.7 m SD) in the restricted state and a mean step length of 214 m (± 256 m SD) in the exploratory state (see Fig. 3 in Appendix for state-specific distributions). Age influenced female movement. The most parsimonious model for state-specific movement for both behavioral states included a two-way interaction between calf fate and age (see Appendix Tables 7 and 8). The Δ estimated—observed step length, regardless of calf fate, was negative in a restricted behavioral state, meaning that female moose moved more than expected once movement was accounted for habitat selection. Older females who had lost a calf moved more than expected compared to females who had not lost a calf (Fig. 3). In an exploratory behavioral state, as they aged, females moved less than expected, regardless of calf fate, once movement was accounted for habitat selection.

### In-season calf survival

**Fig. 4:**
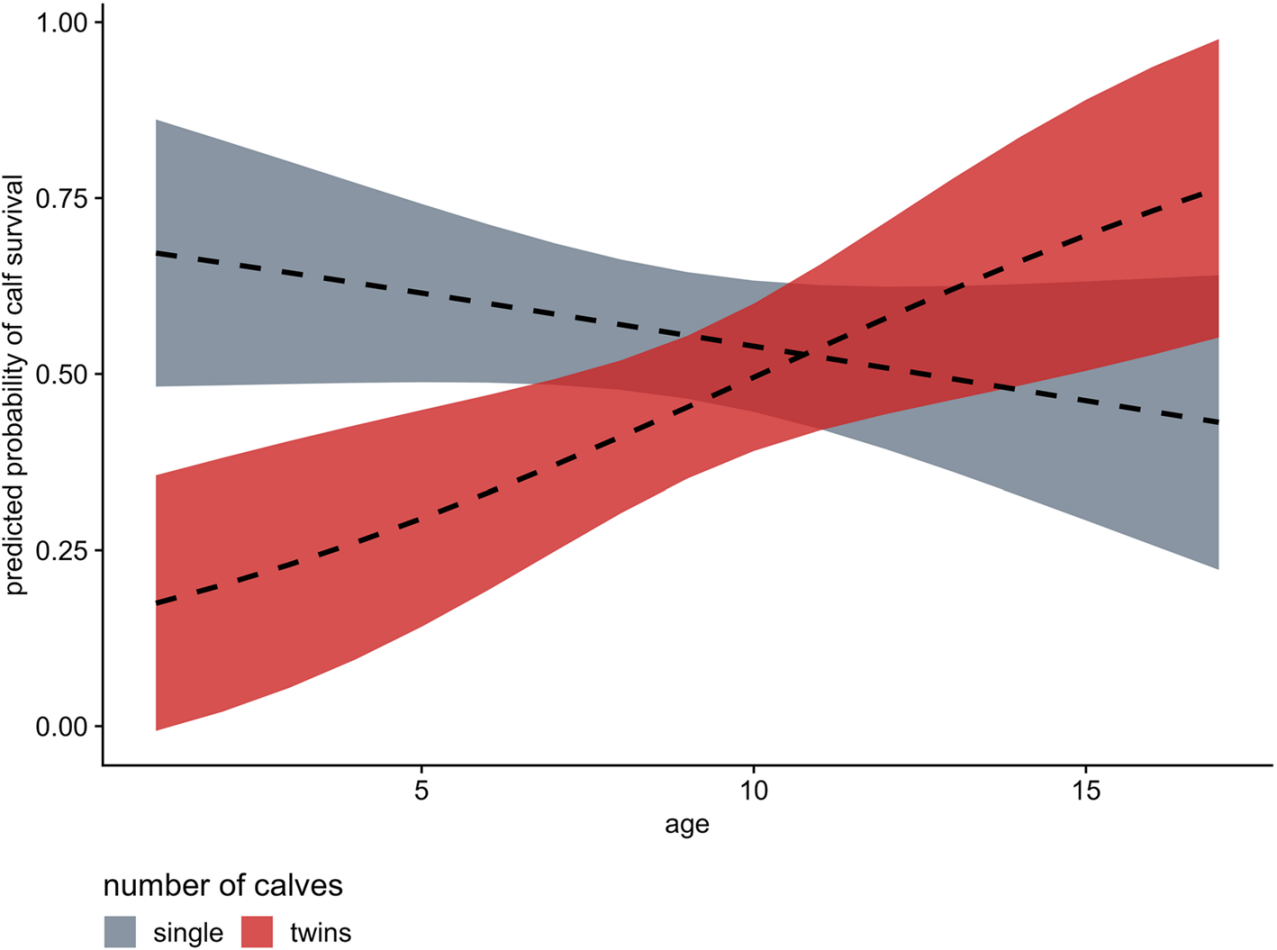
Marginal effects of age and number of calves on calf survival in female moose (n = 76 females in the ongoing hunting season from 2008 to 2018). The red band shows the interaction between age and twins, and the gray band shows the interaction between age and a single calf. Shaded areas reflect the 95% confidence intervals

Age alone had no effect on calf survival in the ongoing hunting season (− 0.062 ± 0.049 SE, *p* = 0.209), indicating that older females were not more successful at avoiding calf harvest during a given season compared to younger females. The number of calves a female had at the onset of the hunting season had a strong negative relationship with offspring survival (− 2.502 ± 0.860 SE, *p* = 0.004), suggesting that females with twins had a greater risk of losing a calf. Age had a positive effect on the survival of multiple calves (0.232 ± 0.088 SE, *p* = 0.008), meaning that older females were more successful at keeping twins alive through an ongoing hunting season (Fig. 4).

## Discussion

In this study, we assessed behavioral changes in a solitary ungulate species in relation to two measures that may indicate experience in reproducing females: calf fate and female age. We derive two overarching conclusions from this study.

### Change in behavior in relation to experience

First, we found that female moose that had lost their calf during the previous hunting season altered their selection for distance to roads and distance to human settlements during the following hunting season. This suggests that the loss of a calf due to human harvest might be a mechanism that induces behavioral change in female moose, supporting our prediction (a) that females will develop non-naive anti-predator behaviors following calf loss. Most noticeably, behavioral adjustments after calf loss were most prevalent during the hunting season and in the restricted behavioral state. Further, we found that previous calf fate often explained the observed behavioral differences in habitat selection and movement better than age (a common proxy for experience). Here, we found that females who had lost a calf in the previous season selected for greater distance to human settlements and lower distance to forests during the period of low hunting pressure. During the day, we found that female moose selected for shorter distance to forest, suggesting they select for more shelter during the hunting season. Likewise, in years following calf loss, they selected for shorter distance to roads during the night, when hunters in Sweden are not allowed to hunt, suggesting a spatiotemporal avoidance of hunters. Similarly, we found that females that had experienced calf loss strongly selected for greater distance to human settlements during the most intense phase of the hunting season. Lastly, we found that older female moose reduced their movement in the restricted behavioral state following calf loss in the previous hunting season, suggesting an adjustment of anti-predator behavior the following year by moving less and thus possibly reducing exposure to human hunters [6, 33]. We interpret these behavioral changes as a possible indication for learning in this solitary ungulate species. However, we cannot rule out that we missed additional behavioral changes in our analysis that could occur in relation to other habitat parameters, such as forage abundance or visibility [78, 79]. We also acknowledge that we considered a proxy for hunting pressure, not fine-scaled hunting pressure statistics such as number of hunters being present in a given area at a given time in relation to moose GPS—locations. Using this proxy likely added additional variation in our analyses, as females likely may not have been exposed to hunting pressure at any given time and place.

### Adjustments of anti-predator behavior in relation to behavioral states

In this study, behavioral change following calf loss was more prevalent in the restricted behavioral state than in the exploratory behavioral state. This finding indicates that when utilizing a limited spatial areal (i.e. the restricted state is defined by shorter steps with low directional persistence, see Fig. 2), female moose select for environmental features that may help them to evade encounters with hunters following harvest of their calf in the previous hunting season. In contrast, in the exploratory behavioral state, we found females to show less behavioral adjustments following calf harvest. Per our definition, this behavioral state includes steps that are longer and more directed. Considering that long scale movements might be necessary in order to avoid human hunters spatially, there might be less room for adjustments of habitat selection or movement in this behavioral state. Additionally, animal responses to mortality risk can be rather short-termed and tortuous, and escapes from human hunters often happen within few minutes [80, 81]. Within this context, it is also important to acknowledge that our analysis was based on 3-h sampling rate of GPS—positions, which might have been too coarse to reflect potential fine-scale adjustments of anti-predator behavior while in the exploratory behavioral state. Alternatively, our chosen environmental covariates and/or their spatial resolution may not have been appropriate to capture the behavioral adjustments in this state. We therefore recommend future studies on behavioral adjustments in relation to experience using more fine-scaled data on hunting pressure, environmental characteristics and at a tighter temporal resolution of GPS—positions.

Our results also suggest that for a female, age and thus likely more experience may result in changed behavioral patterns, supporting our prediction (b) of adjusted behavior over a lifetime. Older female moose selected for habitats providing shelter from human hunters during the hunting season, suggesting that older females counterbalanced the spatiotemporal behavior of human hunters to reduce their harvest risk (i.e., selection for lower distance to forest and selection for greater distance to human settlements), which differed from the selection we observed in younger females. Furthermore, we could link changes in selection for distance to roads, another crucial part of the spatial ecology of human hunters, and the risk of being shot [12, 62] to both calf fate and aging, supporting findings on anti-predator behavior adjustments towards harvest risk in previous research [6, 24]. This suggests an ability to adjust and match behavioral patterns to spatiotemporal variations in harvest risk in a solitary ungulate species like moose. In addition, our results suggest that anti-predator behavior became more refined over time [6, 22, 33]. We want to acknowledge that we could not always link adjusted anti-predator behavior to age or calf fate. This may be due to our study working on a relatively coarse temporal scale (between years and on 3-h time intervals between individual steps where we measured selection). Anti-predator behavior, however, may be expressed at much finer temporal scales (i.e., minutes or seconds) [32, 82]. Lastly, behavioral changes and possible learning processes are complex, emerging from several processes [2, 6, 15], and may occur at points in time not included in this study or may be unrelated to the specific calf loss event that this study addressed. Therefore, performing a similar study in an experimental setting, with detailed information on female behavior and previous experiences, calf fate, and environmental settings could reveal additional information about how anti-predator behavior changes over an individual’s lifetime. Such a setting would help to quantify the impacts of experience on behavior and the adaptive value of the observed behavioral adjustments and would likely remove further unaccounted variation from analyses. Further, it would allow investigating potential accumulation (or the lack thereof) of behavioral change, as these life history parameters are known within such a setting. Within our study setting, we did not know the exact number of calves a female had lost before collaring and our sample size for young females that had yet to reproduce was insufficient to analyze this.

### Calf survival in relation to experience

We did not find evidence that general calf survival was greater in older females, suggesting that even though they may adjust their behavior, the alteration does not have a significant effect on the risk of losing an offspring during a given hunting season. However, older females had a greater chance of keeping twins alive throughout the hunting season, supporting our prediction (c) that age (as a proxy for experience) has an effect on calf survival in an ongoing hunting season. We interpret this as a possible learning effect, perhaps due to accumulated experience in older females, particularly as calves must be shot before their mother.

Environmental variation and degree of sociality influence the need for individual learning as well as opportunities for learning [83]. For individuals in non-gregarious prey species, the number of nonlethal predation events likely are more limited than in gregarious species where individuals can learn, to a greater degree, from the fate of conspecifics (e.g., elk [6], or Indian mynahs (*Acridotheres tristis*) [11]). However, in gregarious species, collection of data on individual offspring loss to human harvest and disentangling relationships between herd members proves to be difficult and require extraordinary amounts of work. In solitary species, this data collection is much simpler since the female–calf relationship is more apparent. Therefore, linking observed behavioral changes in a given female to the survival of her calf is more straightforward.

Previous research has shown that in long-lived species, accumulated experiences allow learning and thus adjustment of behavior over an individuals’ lifetime in relation to changing external conditions, e.g., sea turtles [84] or wandering albatrosses (*Diomedea exulans* [85]). Moose have a lifespan of up to 20 years and may annually reproduce between three and 14 years of age [35, 50]. Therefore, female moose have up to 10 possible learning opportunities (accounting for singletons only; likely more when considering twins), thus allowing for highly adjusted anti-predator behavior.

### Limitations in measuring behavioral responses

Despite largely advancing our understanding of the behavioral ecology of free-ranging animals [86], it is important to note that GPS data do not allow researchers to measure all behavioral adjustments an animal undergoes [87]. For example, some changes in anti-predator behavior are most likely more subtle and expressed on a finer spatiotemporal scale, e.g., short-term tortuous escapes from hunting dogs or decreasing visibility on a local scale by hiding on a minute scale, than the 3-h time interval in this study [88]. Therefore, we may have missed behaviors expressed at finer temporal scales [88]. Moreover, individuals in a non-gregarious species, such as moose, exhibit a considerable degree of variation in movement behavior, likely further increasing variability in anti-predator behavior [89]. We might also have missed behavioral adjustments due to confounding variation between the onset of the hunting season and other ecological factors influencing movement or habitat selection, such as the rutting season. However, this bias applied to all animals and our focus was to investigate the behavioral adjustment at the onset of the hunting season and in relation to diurnal patterns. The use of baying dogs is common for hunting moose in Sweden [47], which may make it difficult even for more experienced individuals to fully undergo the harvest risk. Additionally, the use of hunting dogs and the breed of dogs used may vary among hunting teams and years, which may affect female responses due to different levels of hunting disturbances [80]. Moose may use hiding strategies in the restricted behavioral state that human hunters with baying dogs exploit when releasing the dogs in areas where moose are known to aggregate and track them [80, 88]. Yet, our analyses depart from preys’ perspective, not from the predators. From the preys’ perspective, they still likely aim to expose themselves as little as possible to predators as compared to the explorative behavioral state, which our findings agree on [6, 32, 33].

We look forward to future studies that include data with a higher temporal resolution alongside other data (e.g., acceleration data, heart rate [22, 49, 86, 88]) and perhaps exact habitat quantifiable from cameras on the animal [90] in combination with exact locations and timing of the harvest risk (such as hunters and dogs). Such data may help to detect both stronger and more subtle behavioral changes, and thus increase our understanding of adaptive anti-predator behavior.

## Conclusions

Our study highlights that offspring loss to human hunters induces behavioral change in adult females. This behavioral change may reduce exposure to hunters during high hunting pressure. We therefore recommend that future studies include this information when investigating behavioral change in relation to experience, as it might prove useful to compare direct responses to long-term effects that calf loss to human hunters has on female ungulates.

## Supplementary Information

Additional file1 (DOCX 541 KB)

## Data Availability

Data and code have been archived in the Open Science Framework (OSF) repository and are available via: 10.17605/OSF.IO/NCMZU.

## Abbreviations

HMM: Hidden Markov Model
iSSF: Integrated Step Selection Function
GPS: Global Positioning System
IVW Regression: Inverse variance weighted Regression
