# Supplementary material for: Naivety dies with the calf: calf loss to human hunters imposes behavioral change in a long-lived but heavily harvested ungulate
Source: Mov Ecol. 2024 Sep 23;12:66. doi: 10.1186/s40462-024-00506-5 (PMC11421125; doi:10.1186/s40462-024-00506-5)
Supplement: Supplementary file 1 — Additional file1 (DOCX 541 KB) [file 40462_2024_506_MOESM1_ESM.docx]

Appendix to:

Naivety dies with the calf: Calf loss to human hunters imposes behavioral change in a long-lived but heavily harvested ungulate

Lukas Graf (orcid: [0000-0001-9630-5615](https://orcid.org/0000-0001-9630-5615))^1,2^, Henrik Thurfjell (orcid: [0000-0001-9261-7654](https://orcid.org/0000-0001-9261-7654))^3^, Göran Ericsson (orcid: [0000-0002-5409-7229](https://orcid.org/0000-0002-5409-7229))^1^, Wiebke Neumann (orcid: [0000-0002-0000-4816](https://orcid.org/0000-0002-0000-4816))^1^

^1^Swedish University of Agricultural Sciences, Wildlife, Fish, and Environmental Studies, Skogsmarksgränd, SE-901 83 Umeå, Sweden

^2^Swedish University of Agricultural Sciences, Southern Swedish Forest Research Centre, Sundsvägen 3, SE- 234 22 Lomma, Sweden

^3^SLU Swedish Species Information Centre, Alma allé 8E, 756 51 Uppsala, Sweden

Corresponding author: Lukas Graf (lukas.graf@slu.se)


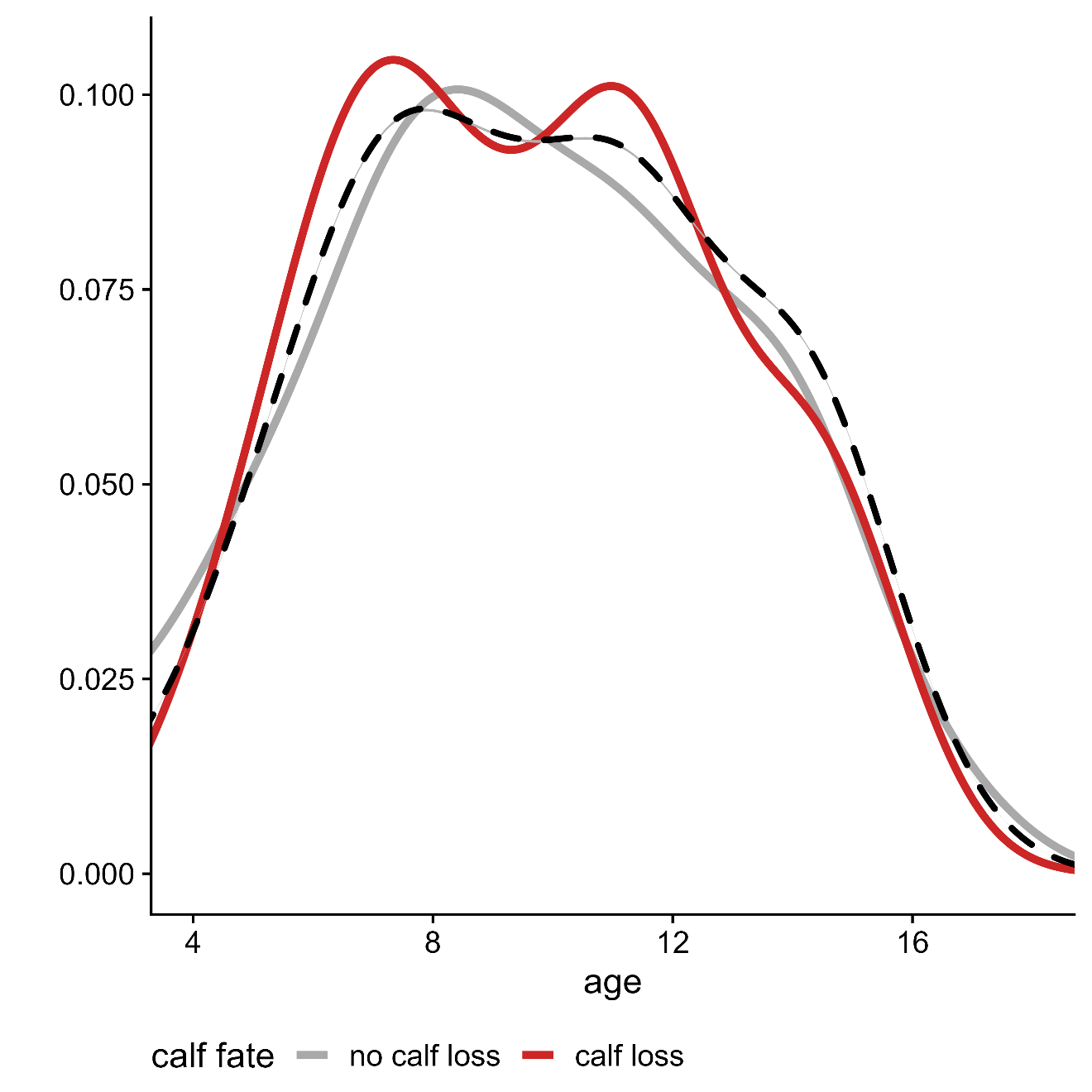


Figure 1 Distributions of calf fate across moose age. The red line shows the distribution of "calf loss", the grey line indicates "no calf loss", respectively. The black dashed line shows the age distribution, free of calf fate.


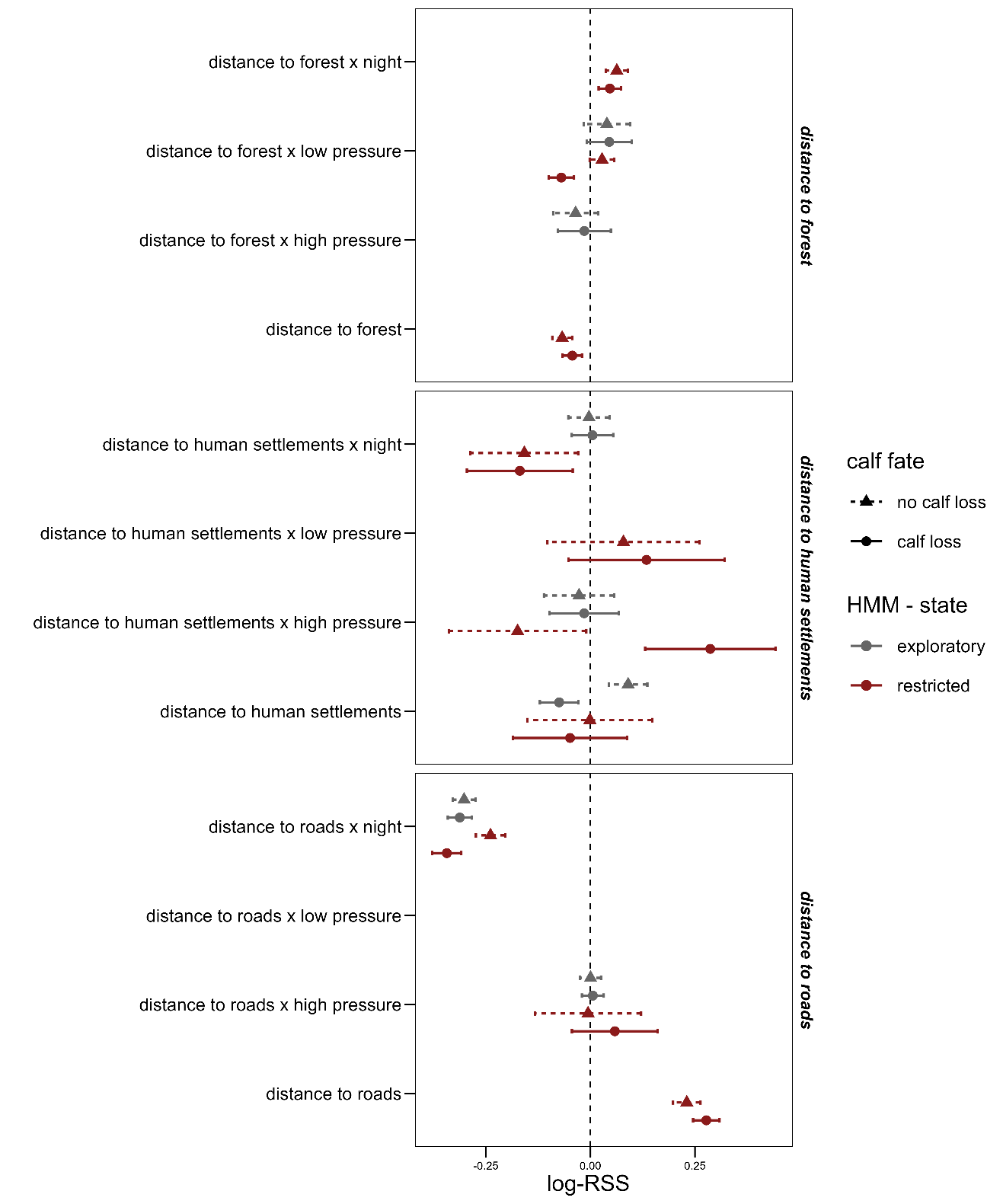


Figure 2 Effects of calf loss through human harvest on female (n = 51 females) habitat selection as estimated by the most parsimonious inverse - variance weighted (IVW) regression (Δ – AICc < 2) that included an effect of calf loss on habitat selection. The vertical dashed line indicates no differences between observed and random steps (i.e., neutral effect) whereas negative values indicate a selection for lower values of environmental predictors and positive values a selection for higher values, respectively. The bars represent the estimated standard errors.


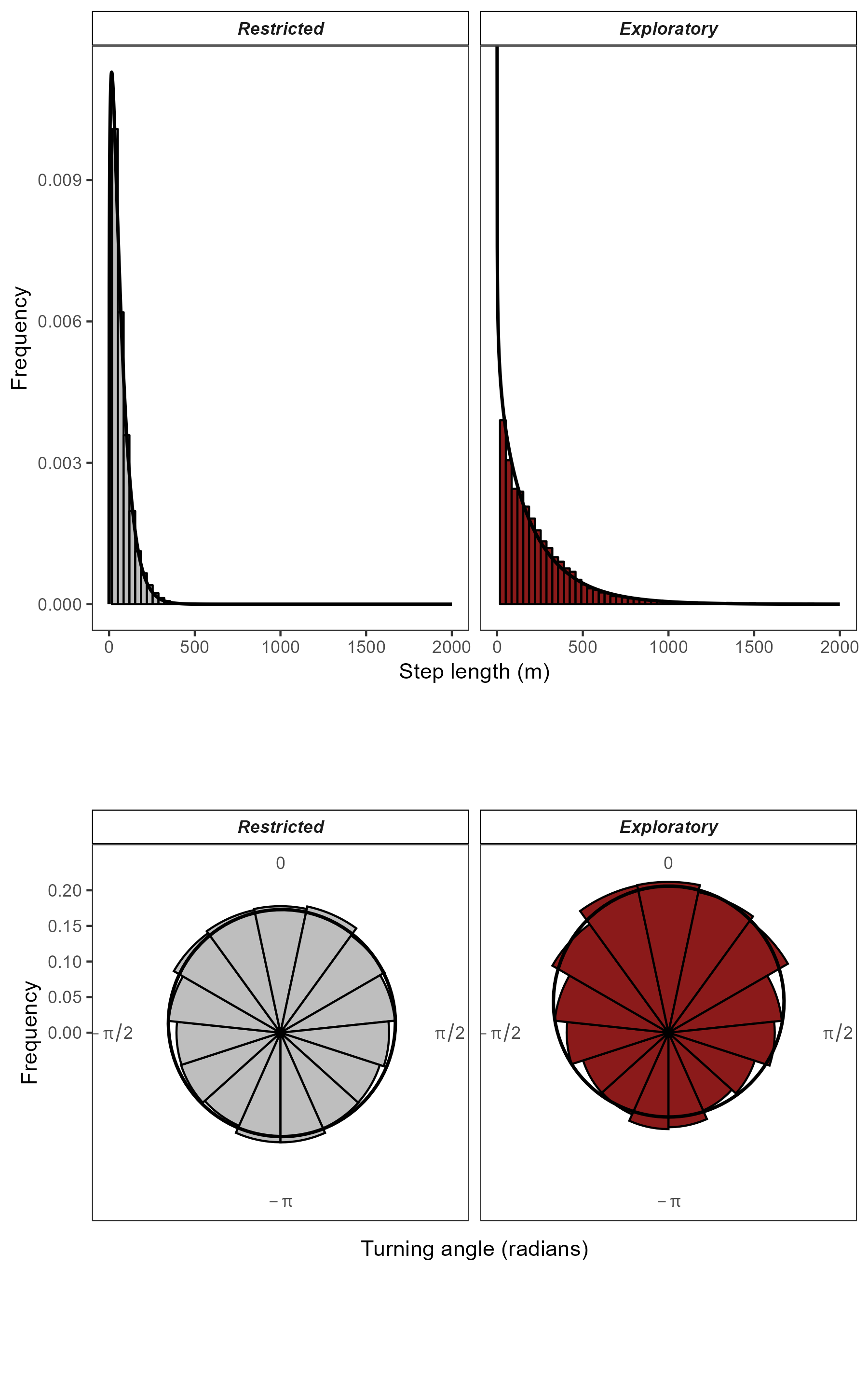


Figure 3 Step length (a) and turning angle (b) of female moose (n = 51) in Växjö and Öster-Malma in an exploratory (right, red) and restricted (left, grey) behavioral state. Black lines show the parametrized distributions of the Hidden Markov Model (HMM). Histograms show raw data in each behavioral state.

Model Selection Part Habitat Selection

We applied generalized linear models (GLMs) with a Gaussian distribution and the inverse variance (1/SE^2^) as weights to test the effects of calf loss (as a learning incentive) and aging on each iSSA coefficient in each state (Picardi et al., 2021; Thurfjell, Ciuti, & Boyce, 2014). We applied four different models to the iSSA coefficients and ranked them using AICc – model selection (Burnham & Anderson, 2002), with a Δ – AIC < 2 to find the most parsimonious model.

Table 1 Model selection tables for distance to humans in an exploratory behavioral state from the inverse - variance weighted regression. We provide the model, log-Likelihood, degrees of freedom (df), AICc, Δ - AICc and AIC - weights. Models are ordered so that the most parsimonious model is at the top.

| **distance to human settlements in exploratory state** | | | | | |
| --- | --- | --- | --- | --- | --- |
| Model | *df* | *logLik* | *AICc* | *Δ-AICc* | *AIC - weight* |
| loss | 3 | -79.38 | 164.96 | 0.00 | 0.78 |
| full | 5 | -79.34 | 169.19 | 4.22 | 0.09 |
| null | 2 | -82.56 | 169.21 | 4.25 | 0.09 |
| age | 3 | -82.49 | 171.17 | 6.21 | 0.04 |

| **distance to human settlements x night in exploratory state** | | | | | |
| --- | --- | --- | --- | --- | --- |
| Model | *df* | *logLik* | *AICc* | *Δ-AICc* | *AIC - weight* |
| null | 2 | -113.20 | 230.50 | 0.00 | 0.56 |
| loss | 3 | -113.14 | 232.47 | 1.97 | 0.21 |
| age | 3 | -113.20 | 232.59 | 2.09 | 0.20 |
| full | 5 | -113.10 | 236.70 | 6.20 | 0.02 |

| **distance to human settlements x high pressure in exploratory state** | | | | | |
| --- | --- | --- | --- | --- | --- |
| Model | *df* | *logLik* | *AICc* | *Δ-AICc* | *AIC - weight* |
| null | 2 | -119.09 | 242.29 | 0.00 | 0.52 |
| loss | 3 | -118.92 | 244.06 | 1.77 | 0.21 |
| age | 3 | -118.99 | 244.19 | 1.91 | 0.20 |
| full | 5 | -117.91 | 246.35 | 4.07 | 0.07 |

| **distance to human settlements x low pressure in exploratory state** | | | | | |
| --- | --- | --- | --- | --- | --- |
| Model | *df* | *logLik* | *AICc* | *Δ-AICc* | *AIC - weight* |
| age | 3 | -102.06 | 210.34 | 0.00 | 0.70 |
| null | 2 | -104.59 | 213.28 | 2.94 | 0.16 |
| full | 5 | -102.02 | 214.61 | 4.27 | 0.08 |
| loss | 3 | -104.59 | 215.40 | 5.05 | 0.06 |

Table 2 Model selection tables for distance to forests in an exploratory behavioral state from the inverse - variance weighted regression. We provide the model, log-Likelihood, degrees of freedom (df), AICc, Δ - AICc and AIC - weights. Models are ordered so that the most parsimonious model is at the top.

| **distance to roads in exploratory state** | | | | | |
| --- | --- | --- | --- | --- | --- |
| Model | *df* | *logLik* | *AICc* | *Δ-AICc* | *AIC - weight* |
| null | 2 | 0.89 | 2.32 | 0.00 | 0.52 |
| age | 3 | 1.21 | 3.79 | 1.46 | 0.25 |
| loss | 3 | 0.89 | 4.42 | 2.10 | 0.18 |
| full | 5 | 1.54 | 7.42 | 5.09 | 0.04 |

| **distance to roads x night in exploratory state** | | | | | |
| --- | --- | --- | --- | --- | --- |
| Model | *df* | *logLik* | *AICc* | *Δ-AICc* | *AIC - weight* |
| null | 2 | -30.59 | 65.29 | 0.00 | 0.54 |
| loss | 3 | -30.34 | 66.88 | 1.60 | 0.24 |
| age | 3 | -30.59 | 67.37 | 2.09 | 0.19 |
| full | 5 | -30.25 | 71.01 | 5.72 | 0.03 |

| **distance to roads x high pressure in exploratory state** | | | | | |
| --- | --- | --- | --- | --- | --- |
| Model | *df* | *logLik* | *AICc* | *Δ-AICc* | *AIC - weight* |
| null | 2 | -26.32 | 56.74 | 0.00 | 0.53 |
| loss | 3 | -26.23 | 58.66 | 1.92 | 0.20 |
| age | 3 | -26.31 | 58.82 | 2.08 | 0.19 |
| full | 5 | -25.09 | 60.70 | 3.96 | 0.07 |

| **distance to roads x low pressure in exploratory state** | | | | | |
| --- | --- | --- | --- | --- | --- |
| Model | *df* | *logLik* | *AICc* | *Δ-AICc* | *AIC - weight* |
| null | 2 | -35.66 | 75.43 | 0.00 | 0.49 |
| age | 3 | -35.08 | 76.38 | 0.95 | 0.30 |
| loss | 3 | -35.65 | 77.53 | 2.10 | 0.17 |
| full | 5 | -34.98 | 80.53 | 5.10 | 0.04 |

Table 3 Model selection tables for distance to forest in an exploratory behavioral state from the inverse - variance weighted regression. We provide the model, log-Likelihood, degrees of freedom (df), AICc, Δ - AICc and AIC - weights. Models are ordered so that the most parsimonious model is at the top.

| **distance to forest in exploratory state** | | | | | |
| --- | --- | --- | --- | --- | --- |
| Model | *df* | *logLik* | *AICc* | *Δ-AICc* | *AIC - weight* |
| null | 2 | -38.58 | 81.27 | 0.00 | 0.48 |
| age | 3 | -37.99 | 82.18 | 0.91 | 0.30 |
| loss | 3 | -38.55 | 83.30 | 2.03 | 0.17 |
| full | 5 | -37.78 | 86.06 | 4.80 | 0.04 |

| **distance to forest x night in exploratory state** | | | | | |
| --- | --- | --- | --- | --- | --- |
| Model | *df* | *logLik* | *AICc* | *Δ-AICc* | *AIC - weight* |
| age | 3 | -43.40 | 93.00 | 0.00 | 0.58 |
| null | 2 | -45.34 | 94.78 | 1.78 | 0.24 |
| full | 5 | -43.02 | 96.55 | 3.55 | 0.10 |
| loss | 3 | -45.25 | 96.71 | 3.71 | 0.09 |

| **distance to forest x high pressure in exploratory state** | | | | | |
| --- | --- | --- | --- | --- | --- |
| Model | *df* | *logLik* | *AICc* | *Δ-AICc* | *AIC - weight* |
| null | 2 | -96.77 | 197.65 | 0.00 | 0.34 |
| loss | 3 | -95.81 | 197.83 | 0.18 | 0.31 |
| age | 3 | -95.88 | 197.97 | 0.31 | 0.29 |
| full | 5 | -95.09 | 200.70 | 3.05 | 0.07 |

| **distance to forest x low pressure in exploratory state** | | | | | |
| --- | --- | --- | --- | --- | --- |
| Model | *df* | *logLik* | *AICc* | *Δ-AICc* | *AIC - weight* |
| null | 2 | -76.25 | 156.62 | 0.00 | 0.45 |
| age | 3 | -75.58 | 157.38 | 0.77 | 0.31 |
| loss | 3 | -76.08 | 158.38 | 1.76 | 0.19 |
| full | 5 | -75.28 | 161.13 | 4.51 | 0.05 |

Table 4 Model selection tables for distance to roads in a restricted behavioral state from the inverse - variance weighted regression. We provide the model, log-Likelihood, degrees of freedom (df), AICc, Δ - AICc and AIC - weights. Models are ordered so that the most parsimonious model is at the top.

| **distance to roads in restricted state** | | | | | |
| --- | --- | --- | --- | --- | --- |
| Model | *df* | *logLik* | *AICc* | *Δ-AICc* | *AIC - weight* |
| loss | 3 | -10.85 | 27.89 | 0.00 | 0.51 |
| null | 2 | -12.42 | 28.94 | 1.04 | 0.30 |
| age | 3 | -12.42 | 31.03 | 3.14 | 0.11 |
| full | 5 | -10.58 | 31.66 | 3.77 | 0.08 |

| **distance to roads x night in restricted state** | | | | | |
| --- | --- | --- | --- | --- | --- |
| Model | *df* | *logLik* | *AICc* | *Δ-AICc* | *AIC - weight* |
| loss | 3 | -27.44 | 61.08 | 0.00 | 0.65 |
| null | 2 | -29.68 | 63.47 | 2.38 | 0.20 |
| full | 5 | -27.32 | 65.13 | 4.05 | 0.09 |
| age | 3 | -29.68 | 65.56 | 4.48 | 0.07 |

| **distance to roads x high pressure in restricted state** | | | | | |
| --- | --- | --- | --- | --- | --- |
| Model | *df* | *logLik* | *AICc* | *Δ-AICc* | *AIC - weight* |
| null | 2 | -66.02 | 136.13 | 0.00 | 0.43 |
| age | 3 | -65.70 | 137.60 | 1.46 | 0.20 |
| full | 5 | -63.55 | 137.61 | 1.47 | 0.20 |
| loss | 3 | -65.91 | 138.02 | 1.89 | 0.16 |

| **distance to roads x low pressure in restricted state** | | | | | |
| --- | --- | --- | --- | --- | --- |
| Model | *df* | *logLik* | *AICc* | *Δ-AICc* | *AIC - weight* |
| null | 2 | -56.43 | 116.96 | 0.00 | 0.56 |
| age | 3 | -56.33 | 118.86 | 1.90 | 0.22 |
| loss | 3 | -56.43 | 119.06 | 2.10 | 0.20 |
| full | 5 | -56.32 | 123.17 | 6.21 | 0.02 |

Table 5 Model selection tables for distance to forest in a restricted behavioral state from the inverse - variance weighted regression. We provide the model, log-Likelihood, degrees of freedom (df), AICc, Δ - AICc and AIC - weights. Models are ordered so that the most parsimonious model is at the top.

| **distance to forest in restricted state** | | | | | |
| --- | --- | --- | --- | --- | --- |
| Model | *df* | *logLik* | *AICc* | *Δ-AICc* | *AIC - weight* |
| loss | 3 | 5.85 | -5.50 | 0.00 | 0.40 |
| null | 2 | 4.77 | -5.45 | 0.05 | 0.38 |
| age | 3 | 4.79 | -3.38 | 2.12 | 0.14 |
| full | 5 | 6.45 | -2.40 | 3.10 | 0.08 |

| **distance to forest x night in restricted state** | | | | | |
| --- | --- | --- | --- | --- | --- |
| Model | *df* | *logLik* | *AICc* | *Δ-AICc* | *AIC - weight* |
| null | 2 | -12.77 | 29.63 | 0.00 | 0.49 |
| loss | 3 | -12.21 | 30.62 | 0.99 | 0.30 |
| age | 3 | -12.77 | 31.73 | 2.10 | 0.17 |
| full | 5 | -12.08 | 34.66 | 5.03 | 0.04 |

| **distance to forest x high pressure in restricted state** | | | | | |
| --- | --- | --- | --- | --- | --- |
| Model | *df* | *logLik* | *AICc* | *Δ-AICc* | *AIC - weight* |
| age | 3 | -45.26 | 96.73 | 0.00 | 0.51 |
| null | 2 | -46.83 | 97.75 | 1.02 | 0.30 |
| loss | 3 | -46.82 | 99.85 | 3.12 | 0.11 |
| full | 5 | -44.93 | 100.38 | 3.65 | 0.08 |

| **distance to forest x low pressure in restricted state** | | | | | |
| --- | --- | --- | --- | --- | --- |
| Model | *df* | *logLik* | *AICc* | *Δ-AICc* | *AIC - weight* |
| loss | 3 | -16.05 | 38.31 | 0.00 | 0.68 |
| null | 2 | -18.69 | 41.47 | 3.16 | 0.14 |
| full | 5 | -15.71 | 41.94 | 3.63 | 0.11 |
| age | 3 | -18.23 | 42.67 | 4.36 | 0.08 |

Table 6 Model selection tables for distance to humans in a restricted behavioral state from the inverse - variance weighted regression. We provide the model, log-Likelihood, degrees of freedom (df), AICc, Δ - AICc and AIC - weights. Models are ordered so that the most parsimonious model is at the top.

| **distance to human settlements in restricted state** | | | | | |
| --- | --- | --- | --- | --- | --- |
| Model | *df* | *logLik* | *AICc* | *Δ-AICc* | *AIC - weight* |
| null | 2 | -171.47 | 347.04 | 0.00 | 0.46 |
| loss | 3 | -170.87 | 347.93 | 0.90 | 0.29 |
| age | 3 | -171.24 | 348.68 | 1.64 | 0.20 |
| full | 5 | -170.50 | 351.50 | 4.46 | 0.05 |

| **distance to human settlements x night in restricted state** | | | | | |
| --- | --- | --- | --- | --- | --- |
| Model | *df* | *logLik* | *AICc* | *Δ-AICc* | *AIC - weight* |
| null | 2 | -167.31 | 338.73 | 0.00 | 0.55 |
| age | 3 | -167.24 | 340.67 | 1.94 | 0.21 |
| loss | 3 | -167.25 | 340.69 | 1.97 | 0.21 |
| full | 5 | -167.00 | 344.51 | 5.78 | 0.03 |

| **distance to human settlements x high pressure in restricted state** | | | | | |
| --- | --- | --- | --- | --- | --- |
| Model | *df* | *logLik* | *AICc* | *Δ-AICc* | *AIC - weight* |
| loss | 3 | -226.92 | 460.04 | 0.00 | 0.60 |
| null | 2 | -228.99 | 462.07 | 2.04 | 0.22 |
| full | 5 | -226.54 | 463.60 | 3.56 | 0.10 |
| age | 3 | -228.84 | 463.88 | 3.85 | 0.09 |

| **distance to human settlements x low pressure in restricted state** | | | | | |
| --- | --- | --- | --- | --- | --- |
| Model | *df* | *logLik* | *AICc* | *Δ-AICc* | *AIC - weight* |
| null | 2 | -203.52 | 411.14 | 0.00 | 0.50 |
| loss | 3 | -203.06 | 412.33 | 1.19 | 0.28 |
| age | 3 | -203.45 | 413.10 | 1.96 | 0.19 |
| full | 5 | -203.03 | 416.58 | 5.44 | 0.03 |

Model Selection Part Movement

We applied linear models (LM) with a Gaussian distribution to test the effects of calf loss (as a learning incentive) and aging on estimated mean step length – observed mean step length. We applied different kind of models four models and rank them using AIC – model selection (Burnham & Anderson, 2002) with a Δ – AIC > 2 to find the most parsimonious model.

Table 7 Model selection tables movement in restricted state. We provide the model, log-Likelihood, degrees of freedom (df), AICc, Δ - AICc and AIC - weights. Models are ordered so that the most parsimonious model is at the top.

| Model | df | logLik | AICc | Δ-AICc | AIC - weight |
| --- | --- | --- | --- | --- | --- |
| full | 5 | -482.16 | 974.81 | 0.00 | 0.83 |
| age | 3 | -486.56 | 979.33 | 4.52 | 0.09 |
| loss | 3 | -486.74 | 979.67 | 4.86 | 0.07 |
| null | 2 | -489.52 | 983.14 | 8.33 | 0.01 |

Table 8 Model selection tables movement in exploratory state. We provide the model, log-Likelihood, degrees of freedom (df), AICc, Δ - AICc and AIC - weights. Models are ordered so that the most parsimonious model is at the top.

| Model | df | logLik | AICc | Δ-AICc | AIC - weight |
| --- | --- | --- | --- | --- | --- |
| full | 5 | -749.20 | 1508.91 | 0.00 | 0.43 |
| null | 2 | -752.97 | 1510.05 | 1.14 | 0.24 |
| loss | 3 | -751.96 | 1510.11 | 1.20 | 0.24 |
| age | 3 | -752.96 | 1512.11 | 3.20 | 0.09 |

References:

Burnham, K. P., & Anderson, D. R. (2002). A practical information-theoretic approach. *Model selection and multimodel inference, 2nd ed. Springer, New York, 2*.

Picardi, S., Coates, P., Kolar, J., O'Neil, S., Mathews, S., & Dahlgren, D. (2021). Behavioural state‐dependent habitat selection and implications for animal translocations. *Journal of Applied Ecology, 59*(2), 624-635. doi:10.1111/1365-2664.14080

Thurfjell, H., Ciuti, S., & Boyce, M. S. (2014). Applications of step-selection functions in ecology and conservation. *Mov Ecol, 2*(1), 4. doi:10.1186/2051-3933-2-4
